# Development of Electrochemical Sensor Using Iron (III) Phthalocyanine/Gold Nanoparticle/Graphene Hybrid Film for Highly Selective Determination of Nicotine in Human Salivary Samples

**DOI:** 10.3390/bios13090839

**Published:** 2023-08-23

**Authors:** Kavitha Kamalasekaran, Vasanth Magesh, Raji Atchudan, Sandeep Arya, Ashok K. Sundramoorthy

**Affiliations:** 1 Department of Chemistry, Velammal Engineering College, Chennai 600066, Tamil Nadu, India; kavithak@velammal.edu.in; 2 Centre for Nano-Biosensors, Department of Prosthodontics, Saveetha Institute of Medical and Technical Sciences, Saveetha Dental College and Hospitals, Poonamallee High Road, Velappanchavadi, Chennai 600077, Tamil Nadu, India; jamesvasanth313@gmail.com; 3 School of Chemical Engineering, Yeungnam University, Gyeongsan 38541, Republic of Korea; atchudanr@gmail.com; 4 Department of Physics, University of Jammu, Jammu 180006, Jammu and Kashmir, India; snp09arya@gmail.com

**Keywords:** nicotine determination, iron phthalocyanine, modified electrode, graphene sensor

## Abstract

Nicotine is the one of the major addictive substances; the overdose of nicotine (NIC) consumption causes increasing heart rate, blood pressure, stroke, lung cancer, and respiratory illnesses. In this study, we have developed a precise and sensitive electrochemical sensor for nicotine detection in saliva samples. It was built on a glassy carbon electrode (GCE) modified with graphene (Gr), iron (III) phthalocyanine-4,4′,4″,4′′′-tetrasulfonic acid (Fe(III)Pc), and gold nanoparticles (AuNPs/Fe(III)Pc/Gr/GCE). The AuNPs/Fe(III)Pc/Gr nanocomposite was prepared and characterized by using FE-SEM, EDX, and E-mapping techniques to confirm the composite formation as well as the even distribution of elements. Furthermore, the newly prepared AuNPs/Fe(III)Pc/Gr/GCE-nanocomposite-based sensor was used to detect the nicotine in phosphate-buffered solution (0.1 M PBS, pH 7.4). The AuNPs/Fe(III)Pc/Gr/GCE-based sensor offered a linear response against NIC from 0.5 to 27 µM with a limit of detection (LOD) of 17 nM using the amperometry (i–t curve) technique. This electrochemical sensor demonstrated astounding selectivity and sensitivity during NIC detection in the presence of common interfering molecules in 0.1 M PBS. Moreover, the effect of pH on NIC electro-oxidation was studied, which indicated that PBS with pH 7.4 was the best medium for NIC determination. Finally, the AuNPs/Fe(III)Pc/Gr/GCE sensor was used to accurately determine NIC concentration in human saliva samples, and the recovery percentages were also calculated.

## 1. Introduction

**Figure 1:**
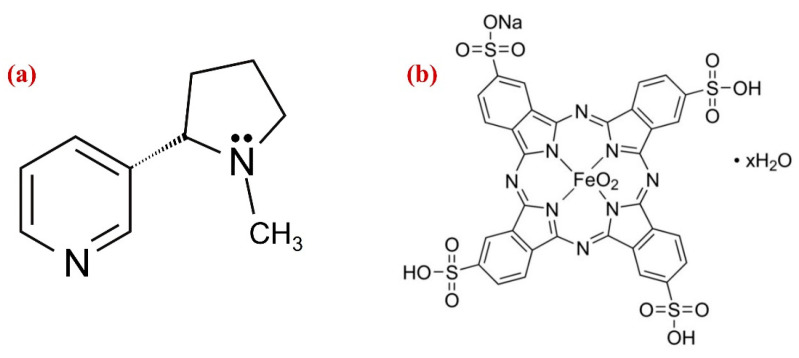
Chemical structures of (**a**) nicotine and (**b**) iron (III) phthalocyanine-4,4′,4″,4′′′-tetrasulfonic acid (Fe(III)Pc).

Nicotine use is a global epidemic among young people; it is a highly addictive substance found in tobacco plants (Figure 1a). The primary mechanism by which NIC affects the cardiovascular system is by stimulating the sympathetic nervous system, which causes norepinephrine to be released, increasing the heart rate, blood pressure, cardiac contractility, and systemic vasoconstriction [1]. Regular consumption of NIC has a number of potentially dangerous diseases and effects on human health, including elevated blood pressure and heart rate, slowed healing times, hypertension, and vasoconstriction, which can contribute to cardiovascular disease [2]. NIC affects the central nervous system by elevating mood, providing a sense of satisfaction, and reviving energy [3]. For adults, the effective dosage of NIC is around 40–60 mg, while the dose for children is ~10 mg [4,5]. Despite nicotine’s significant toxicity, the research has shown that it may have therapeutic benefits for the treatment of Parkinson’s and Alzheimer’s diseases [6]. As a result, determination of NIC concentration in tobacco items and medical samples is crucial for medical diagnostics [7,8].

Due to its importance, several analytical methods for the accurate and rapid determination of NIC in cigarettes, chewing gum, food products, and numerous biological samples like blood serum, saliva, sweat, and urine have been developed. All these reported methods have involved with different analytical protocols such as radioimmunoassay [9], spectrofluorimetric methods [10], capillary electrophoresis [11], gas chromatography-mass spectrometry (GC-MS) [12], spectrophotometry [13,14], and high-performance liquid chromatography (HPLC) [15,16]. Although these reported methods had produced excellent performances towards NIC determination, they are generally inconvenient, more expensive, associated with difficult sample pre-treatment processes involving the extraction of NIC from the sample matrix and also need of skilled operators to perform the experiment with the utmost care. Additionally, the demand for highly skilled workers frequently limits their utilization in regular analytical practice.

On the other hand, electrochemical-based techniques have been discovered to be more advantageous compared with other methods because of their low cost and compactness with portable and quick detection [17,18,19,20,21,22]. However, the typical commercially available electrodes were not sensitive enough to detect NIC at low concentrations. In order to increase the sensor’s selectivity and sensitivity, electrode-surface modification was used as a technique [23,24]. Recently, researchers have employed carbon paste sensors adapted with nano-TiO_2_ [1], multiwalled carbon nanotube/graphene composites [25], cerium-nanoparticle-coated carbon paste sensors [26], carbon composite electrodes (CPEs) based on hydrogen titanate [27], boron-doped diamond electrodes [28,29], and silver-nanoparticle-modified electrodes. Some of these methods used multistep preparation processes, expensive chemicals and reagents, and the electrodes also underwent degradation and showed poor stability.

The choice of a suitable and affordable electrode modification material that can act as an electrocatalyst on the electrodes’ surface is the main challenge in the construction of an electrochemical sensor. Graphene (Gr) is an allotrope of carbon with a two-dimensional (2D) lattice nanostructure with a delocalized electronic network [30,31,32,33,34]. Researchers have paid greater attention towards graphene due to its strong mechanical strength, large surface area, excellent thermal conductivity, optical transparency, and good chemical tolerance for protective coatings [35,36,37]. To be specific, the surface confined functional groups present on the graphene provided support and compatibility with other nanoparticles to improve the sensor’s selectivity and sensitivity in the electrocatalytic reaction [38]. Generally, Gr can be prepared either by a bottom-up process or a top-down method [39,40,41,42]. In recent years, Gr nanosheets were prepared by the electro-chemical exfoliation method with a high yield at a low cost [43,44,45,46].

On the other hand, metallopthalocyanine (MPc), which has a robust 18-conjugated system and a high electron density was considered as an excellent choice to use as an electron transfer mediator [47]. MPc comprising transition metal ions such as Fe(II), Fe(III), Co(III), Co(II), Cu(II), Cr(III) and Zn(II) exhibit highly active redox pairs under specific circumstances, which helped several molecules undergo oxidation or reduction. The influence of the ligand and its substituent can be used to tune the electronic state of MPc’s metallic centers. As a result, catalytic abilities of the complex can be enhanced, and the coordination setting made it possible for specific analyte to be adsorbed to the system, increasing its specificity [48]. The immobilization of the complexes in a composite is a clever strategy to deal with this circumstance. Because of the non-covalent stacking interactions between the porphyrin rings and the aromatic rings of the carbon-based nanomaterials, MPc was commonly used to immobilize the nanomaterials [49]. Among all carbon nanomaterials, the 2D Gr was preferred for making effective electrochemical sensors due to its broad electrochemical window, chemical inertness, thermal stability, and good charge carrier mobility [50], and also metal nanoparticles (Au, Ag, Pd and Pt) have numerous benefits, including their substantial surface area, excellent biocompatibility, high conductivity, renewable surface, and electrocatalytic activities [51,52,53,54]. Although, the AuNPs have been employed previously to prepare a variety of modified electrodes, in this study, for the first time, iron (III) phthalocyanine-4,4′,4″,4′′′-tetrasulfonic acid (Fe(III)Pc) (Figure 1b) integrated with AuNPs/Gr has been used to construct an electrochemical sensor for the determination of NIC concentration in human saliva.

## 2. Experimental

### 2.1. Materials and Reagents

Graphite was obtained from Graphite Store, Inc. (United States). Iron (III) phthalocyanine-4,4′,4″,4′′′-tetrasulfonic acid (Dye content 80%), sparfloxacin (purity 98%), and nicotine (purity >99%) were purchased from Sigma-Aldrich, India. Gold (III) chloride trihydrate (Tetrachloroauric acid) (HauCl_4_·3H_2_O), acetic acid, dopamine, hydrogen peroxide (H_2_O_2_), magnesium chloride (MgCl_2_), and ascorbic acid were acquired from Sisco Research Laboratories (SRL) Pvt. Ltd., India. Sodium phosphate dibasic heptahydrate (Na_2_HPO_4_·7H_2_O) and sodium dihydrogen phosphate monohydrate (H_2_NaPO_4_·H_2_O) were purchased from Spectrochem Pvt, Ltd., India. Calcium chloride (CaCl_2_) was obtained from SD Fine-Chem Ltd., Mumbai, India. Hydrochloric acid (HCl), dextrose (glucose), sodium chloride (NaCl), and sodium hydroxide were obtained from Merck, India. All the chemicals were used without any additional purification. Nicotine solution was prepared using double distilled water (Milli-Q water) (18.2 MΩ cm) and stored under dark conditions. Other necessary solutions and buffers have been prepared using the regular laboratory procedures.

### 2.2. Characterization

The surface morphologies of the Gr, Fe(III)Pc/Gr, and AuNPs/Fe(III)Pc/Gr composite were investigated with field-emission scanning electron microscope (FE-SEM, JSM IT800 (JEOL, Tokyo, Japan)). The energy dispersive X-ray spectrum (EDS) and elemental mapping (E-Map) data were successfully recorded with XPLORE-30 (Oxford, UK). A CHI-760E electrochemical workstation (CH Instruments, Bee Cave, TX, USA) with a three-electrode system consisting of a counter electrode (Pt wire), a reference electrode (Ag/AgCl immersed in 3 M KCl), and a working electrode (GCE with a 0.07 cm^2^ working area) were used to conduct the electrochemical measurements such as cyclic voltammetry (CV), electrochemical impedance spectroscopy (EIS), differential pulse voltammetry (DPV), and amperometry (i–t curve).

### 2.3. Preparation of Graphene Dispersion

Initially, Gr was synthesized by electrochemical exfoliation method [55,56]. In the electrochemical cell, platinum wire as the counter electrode (cathode) and a graphite (99% purity) electrode was used as the working electrode (anode). The 0.1 M phosphate-buffered solution was used as electrolyte in the electrochemical cell. Using a DC power source, +10 V electric potential was applied across the electrodes for 30 min at room temperature (RT) to exfoliate the graphene sheets. In order to peel out graphene sheets, an anodic potential oxidized the graphite rod and allowed ionic species in the electrolyte to enter through the interlayer distance of the graphite. Finally, the graphene sheets were peeled off during the exfoliation process and settled down in PBS. These exfoliated graphene flakes were collected and repeatedly cleaned with de-ionized (DI) water to remove the ionic species using Whatman filter paper, then the pure graphene powder was collected. Subsequently, the obtained graphene sheets were probe sonicated in 30 mL distilled water for 30 min at RT.

### 2.4. Chemical Synthesis of Gold Nanoparticles (AuNPs)

The AuNPs were synthesized by chemical reduction method. In brief, the 10 mg gold (III) chloride trihydrate (HAuCl_4_·3H_2_O) was dissolved in 10 mL distilled water. Then, 4 mL of ethanol was added to the gold (III) chloride solution and stirred (at 500 rpm) using a magnetic stirrer for 5 min at 60 °C (transparent yellow color solution appeared). After that, 3 mL of 2 mM sparfloxacin (SFC) solution prepared in 0.1 M HCl was added in to the solution mixture and stirred (500 rpm) at 60 °C for 5 min. After the addition of SFC, the transparent yellow color of the solution changed into glitter goldish-yellow color. Next, 0.2 mL of 0.5 M NaOH was added drop wise with constant stirring at 60 °C. After the addition of few drops of NaOH, goldish-yellow color solution was changed into red-wine color, which confirmed the formation of AuNPs. After that, the solution was cooled down to RT and the supernatant (yellow color) and precipitate (red color) were separated by centrifugation (15 mL centrifuge tube) at 3000 rpm for 15 min. To the precipitate (red), 5 mL of distilled water was added and dispersed well. A reddish-pink color dispersion was obtained, which indicated the formation of stable AuNPs colloidal solution. The supernatant was subjected to further characterization and use.

### 2.5. Synthesis of Graphene/Fe(III)Pc/AuNPs Composite

The 5 mg of Fe(III)Pc was dissolved in 10 mL of distilled water. Then, the Gr dispersion and Fe(III)Pc solution were mixed together at 10:2 ratio (10 mL of Gr dispersed solution and 2 mL of Fe(III)Pc) with the help of ultrasonic bath sonicator for 10 min under RT. After that, AuNPs colloidal solution was added in to the Fe(III)Pc/Gr mixture at 1:1 ratio and bath sonicated for 10 min at RT. This prepared composite dispersion was later used for the modification of GCE to determine nicotine concentration.

### 2.6. Preparation of AuNPs/Fe(III)Pc/Gr/GCE Sensor

Firstly, the working electrode surface was cleaned with 0.05 µm alumina slurry and then rinsed with distilled water. Then, 7 µL of AuNPs/Fe(III)Pc/Gr dispersion was drop casted on to the GCE’s surface and heated at 50 °C in a hot air oven. To remove the unbounded materials from the electrode surface, the AuNPs/Fe(III)Pc/Gr/GCE was gently immersed in distilled water and stored it for further use. For the control studies, Gr/GCE and Fe(III)Pc/Gr/GCE (without AuNPs) modified electrodes were also prepared in the same way.

### 2.7. Preparation of Different pH Buffer Solutions

The 0.1 M PBS solution was prepared with different pH values by adjusting the ratio of Na_2_HPO_4_.7H_2_O and H_2_NaPO_4_.H_2_O. The ratio was adjusted to obtain pH values of 6, 7.4, and 8. In addition, pH 2 and 4 were prepared by adjusting the pH 6 0.1 M PBS with 1 M HCl, whereas pH 10 was prepared by adjusting the pH 8 0.1 M PBS with 1 M NaOH.

### 2.8. Real-World Sample Collection and Analysis

Salivary samples were collected in early morning (6:30 a.m.) from a person (age = 62), who frequently used tabaco products. The informed consent form from the volunteer who provided saliva sample was obtained with the standard protocol as per the institution norms. To eliminate the macro-molecules and debris, the obtained saliva sample was diluted with DI water at a 1:2 ratio (5 mL saliva and 10 mL DI water) and centrifuged for 5 min at 3000 rpm. The supernatant was separated following the centrifugation and kept at 4 °C. The saliva sample was tested using AuNPs/Fe(III)Pc/Gr/GCE to determine nicotine concentration.

## 3. Results and Discussion

### 3.1. Material Characterizations (FESEM, EDS and E-Mapping)

**Figure 2:**
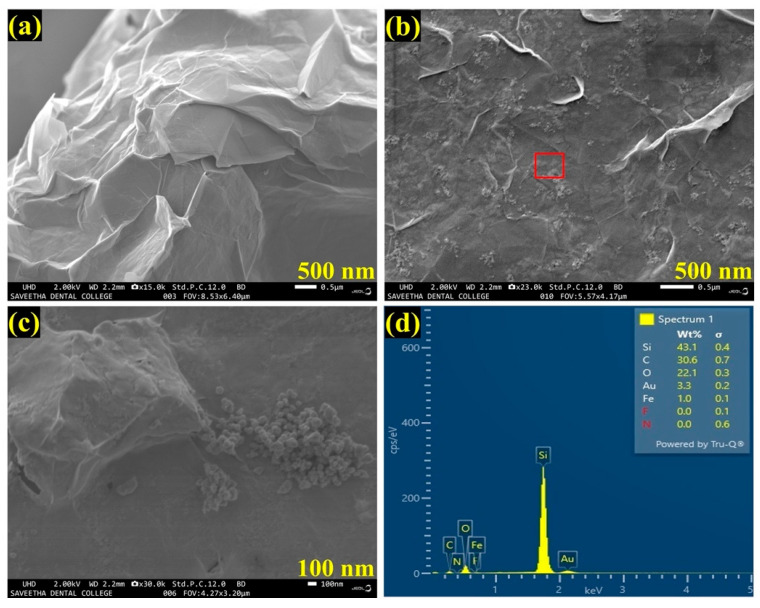
FESEM images of (**a**) Gr and (**b**) AuNPs/Fe(III)Pc/Gr composite, and (**c**) the highly magnified portion of the highlighted area in image **b**, which shows the presence of AuNPs on Gr surface. (**d**) EDS spectrum of AuNPs/Fe(III)Pc/Gr composite.

The FESEM used to analyze the structural morphology of the synthesized materials. As shown in Figure 2a, an FESEM image of as-synthesized Gr had clearly indicated the successful synthesis of few-layered Gr with wrinkles on the layers. Further, an FESEM image of the AuNPs/Fe(III)Pc/Gr composite exhibited the successful integration of AuNPs on the Fe(III)Pc/Gr surface (Figure 2b). With the 100 nm resolution, Figure 2c showed the highly magnified portion of Figure 2b, that confirmed the presence of the composite material (Fe(III)Pc/Gr and AuNPs). In addition, the presence of AuNPs on the Fe(III)Pc/Gr surface was also clearly observed by the FESEM image analysis.

**Figure 3:**
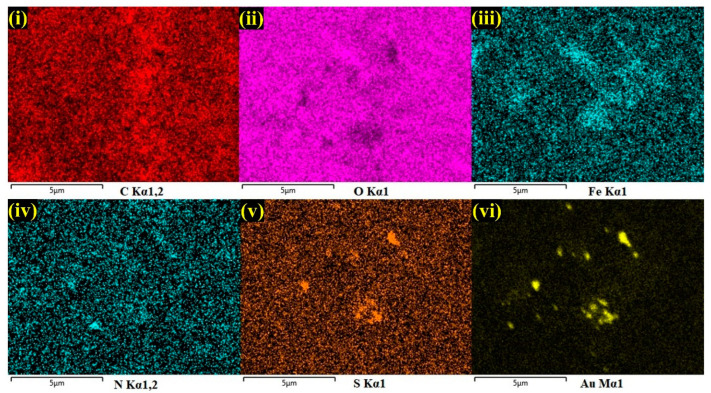
E-mapping analysis of AuNPs/Fe(III)Pc/Gr composite: (**i**) C, (**ii**) O, (**iii**) Fe, (**iv**) N, (**v**) S, and (**vi**) Au.

Furthermore, by taking measurements over the randomly selected area, EDS spectrum was recorded, which indicated the presences of carbon (C-30.6%), oxygen (O-22.1%), iron (Fe-1.0%), and gold (Au-3.3%), which demonstrated the effective formation of the AuNPs/Fe(III)Pc/Gr composite film (Figure 2d). The silicon (Si-43.1%) peak was also observed in the EDS spectrum, which was due to usage of the Si substrate for the sample preparation. The E-mapping of the sample had also confirmed the homogenous distribution of all the elements on the AuNPs/Fe(III)Pc/Gr composite, as shown in Figure 3. In E-mapping images, the presence of (image i) C and (image ii) O indicated the presence of Gr. Similarly, the (image iii) Fe, (image iv) N (Nitrogen), and (image v) S (sulfur) content clearly indicated the presence of the Fe(III)Pc compound in the composite material. Also, the elemental mapping image (vi) of Au revealed the uniform distribution of AuNPs on the composite film (Figure 3).

### 3.2. Electrochemical Properties of AuNPs/Fe(III)Pc/Gr Composite

**Figure 4:**
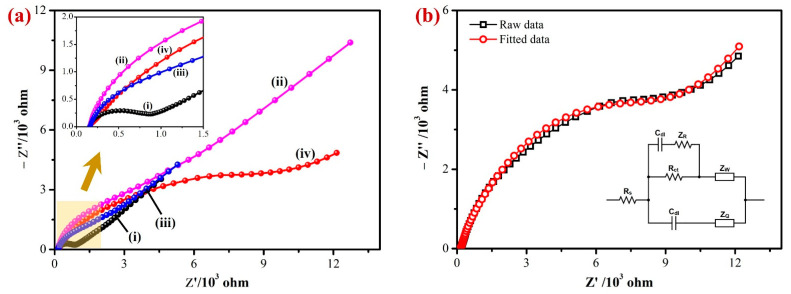
(**a**) The EIS data of the (i) bare/GCE, (ii) Gr/GCE, (iii) Fe(III)Pc/Gr/GCE, and (iv) AuNPs/Fe(III)Pc/Gr/GCE’s were recorded at an amplitude of 5 mV (0.1 to 10^4^ Hz) in 0.1 M KCl with 2 mM [Fe(CN)_6_]^3−^. Inset: The highlighted area on Figure 4a was magnified, as it can be seen that the modified electrodes (Gr/GCE, Fe(III)Pc/Gr/GCE, and AuNPs/Fe(III)Pc/Gr/GCE) did not display semicircles at higher frequencies. (**b**) EIS data of AuNPs/Fe(III)Pc/Gr/GCE (black curve) were fitted with the equivalent circuit model (red curve). Inset: Randle’s circuit diagram.

Electrochemical impedance spectroscopy (EIS) was used to examine the interfacial electron transfer resistance (R_ct_) of the developed sensor. Figure 4a shows the Nyquist plots obtained for the bare/GCE (i), Gr/GCE (ii), Fe(III)Pc/Gr/GCE (iii), and AuNPs/Fe(III)Pc/Gr/GCE’s (iv) in 0.1 M KCl containing 2 mM [Fe(CN)_6_]^3−^ [57]. The charge transfer resistance (R_ct_) values for each modified electrode were calculated based on the semicircle’s diameter observed in the high-frequency area of the Nyquist plots. In the Nyquist plot, the bare GCE showed a semicircle with an R_ct_ value of 654 Ω, indicated the highest electron transfer resistance among the tested electrodes. In comparison to the bare GCE, all the modified electrodes did not show any semicircles at higher frequencies due to a lower electron transfer resistance (inset of Figure 4a).

The diameter of the semicircle was significantly reduced when the graphene was immobilized on the GCE surface. This was because graphene had a high electrical conductivity, which lowered the GCE’s impedance. The imaginary impedance of Fe(III)Pc/Gr/GCE had been determined to be lower than that of Gr/GCE but greater than that of bare GCE in the higher frequency range. This reduction in the impedance can be attributed to the considerably lower conductivity of Fe(III)Pc compared to Gr, because of the distinct chemical environment around Fe(III)Pc [58]. It is important to note that there was not an obvious semicircle, which can be related to the existence of Gr in the electrode configuration. The Nyquist plots of AuNPs/Fe(III)Pc/Gr/GCE had a higher R_ct_ (8611 Ω) than the bare GCE (654 Ω) and modified GCE (Fe(III)Pc/Gr/GCE—1108 Ω). This was possibly due to the negatively charged composite present on the GCE that repelled negatively charged [Fe(CN)_6_]^3−^ ions (Figure 4b). The Randles’s equivalent circuit model was created according to the AuNPs/Fe(III)Pc/Gr/GCE data which were fitted with 99.98% accuracy (inset of Figure 4b). To fully comprehend this conclusion, three major factors must be considered: electrostatic repulsion (i), steric hindrance (ii), and improvement in the electrochemical kinetics of the redox marker reaction (iii). It is generally recognized that modifying the electrode surface has a major impact on the electron transfer process of [Fe(CN)_6_]^3−^, which was consistent with the earlier findings [59,60]. The initial rise in R_ct_ can be explained based on the electrostatic repulsion between species bearing the same charge, when [Fe(CN)_6_]^3−^ was utilized. Furthermore, the presence of AuNPs on the electrode surface may provide a steric barrier, further contributing to an increase in Rct. Both of these variables contributed to the overall increase in the charge transfer resistance. Further, a relatively higher impedance was observed in 2 mM [Fe(CN)_6_]^3−^, when the electrode was modified with the AuNPs/Fe(III)Pc/Gr, which suggested that the reactive composite was stable with negatively charged functional groups (Figure 4b) [61].

**Figure 5:**
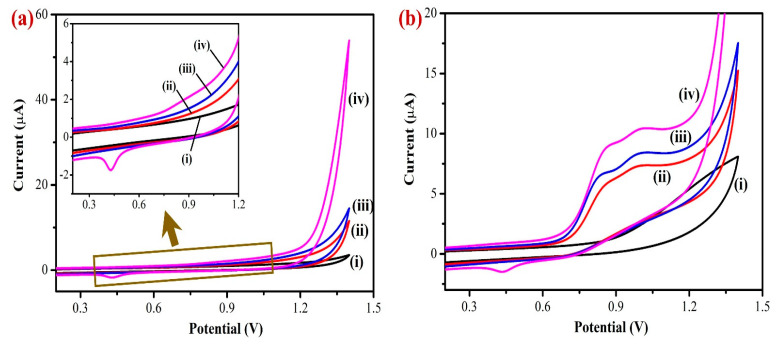
Cyclic voltammograms (CVs) of (i) bare/GCE, (ii) Gr/GCE, (iii) Fe(III)Pc/Gr/GCE, and (iv) AuNPs/Fe(III)Pc/Gr/GCE in 0.1 M PBS (pH = 7.4): (**a**) without and (**b**) with the addition of 400 µM NIC at a scan rate 50 mV s^−1^. Inset of Figure 5a shows the enlarged CV curve of AuNPs/Fe(III)Pc/Gr/GCE that exhibited a redox peak of AuNPs (E_pa_ at 0.8 V and E_pc_ at 0.4 V).

Next, cyclic voltammetry (CV) was used to measure the electrochemical activity of the modified electrodes in PBS (0.1 M, pH 7.4). The electrochemical properties of the bare GCE, Gr/GCE, Fe(III)Pc/Gr/GCE, and AuNPs/Fe(III)Pc/Gr/GCE were tested in PBS (0.1 M pH 7.4) without analyte (nicotine) at a scan rate of 50 mV s^−1^ (Figure 5a). Compared to bare GCE, the background current intensities of Gr/GCE and Fe(III)Pc/Gr/GCE increased progressively after the modification. But, a high capacitance CV was observed for the AuNPs/Fe(III)Pc/Gr/GCE compared to other electrodes, which suggested the presence of a higher electroactive surface area brought on by the composite material. In addition, the intricate structure of the composite resulted in higher anodic and cathodic peak currents due to redox activity of AuNPs (Figure 5a, curve iv).

The electro-catalytic activities of the modified and unmodified electrodes were investigated in PBS (0.1 M) containing 400 µM NIC at a scan rate of 50 mV s^−1^ (Figure 5b). In this study, the unmodified electrode (bare GCE) showed the lowest electrochemical oxidation peak current for NIC at 1.2 V. The Gr/GCE had shifted the oxidation peak of NIC from 1.2 V to 0.85 V and the Fe(III)Pc/Gr/GCE had shown a considerable higher oxidation peak current than Gr/GCE for NIC, which was due to the presence of Fe(III)Pc co-ordination complex that increased the electro-catalytic activity of the composite modified electrode (Figure 5b, curves i–iv).

Moreover, AuNPs/Fe(III)Pc/Gr/GCE showed a strong electrocatalytic property, which led to a very high irreversible oxidation peak for nicotine at 0.85 V. It may be due to the presence of AuNPs on the composite material that highly enhanced the electrocatalytic activity of the sensor towards NIC oxidation. The AuNPs/Fe(III)Pc/Gr/GCE showed high electrocatalytic activity for NIC oxidation at a lower potential (0.85 V) with a higher peak current (9.1 µA). This enhanced electrocatalytic activity might have resulted due to the high surface-to-volume ratio of the AuNPs/Fe(III)Pc/Gr composite material, which also contributed to a good electron transfer reaction between NIC and the AuNPs/Fe(III)Pc/Gr/GCE.

### 3.3. Determination of NIC by CV

**Figure 6:**
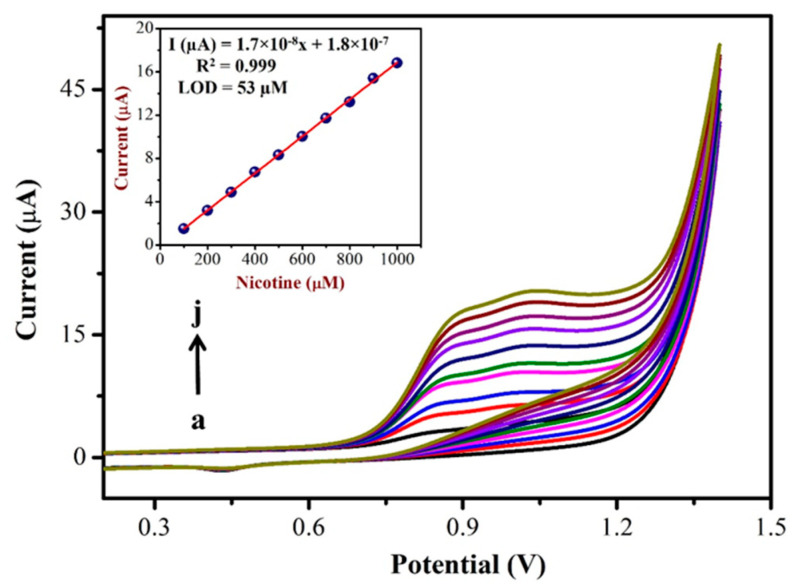
CVs were recorded with various concentrations of NIC from (a) 100 µM to (j) 1 mM using AuNPs/Fe(III)Pc/Gr/GCE in 0.1 M PBS (pH = 7.4) at a scan rate of 50 mV s^−1^. Inset: The calibration curve was made between various concentrations of [NIC] and I_pa_ of NIC (µA) (the blank current was subtracted from the NIC oxidation peak current for each concentration).

To prepare a calibration curve, CVs were recorded using a AuNPs/Fe(III)Pc/Gr/GCE with different concentrations of NIC. Figure 6 shows the CVs obtained for the addition of various NIC concentrations in the range of 100 µM to 1 mM in PBS (0.1 M, pH = 7.4). In this study, after the addition of each concentration of NIC (in the interval of 100 µM), CVs were recorded. The oxidation peak currents of NIC linearly increased with respect to the increasing concentration of NIC, which had indicated the high sensitivity of AuNPs/ Fe(III)Pc/Gr/GCE for NIC oxidation. Figure 6 (Inset) also shows the linear relationship between the peak currents and various concentrations of NIC. As shown, the oxidation peak current (at 0.85 V) increased linearly with respect to the NIC concentration with a linear regression equation and R^2^ value of Y = 1.7 × 10^−8^x + 1.8 × 10^−7^ and R^2^ = 0.999, respectively. The limit of detection (LOD) for NIC was calculated as 53 µM using the CV data and was determined using Equation (1), where ‘S’ denotes the slope of the calibration curve, while σ denotes the standard deviation of the response [62].

LOD = 3.3 × σ/S(1)

### 3.4. Impact of Scan Rate on NIC Oxidation

**Figure 7:**
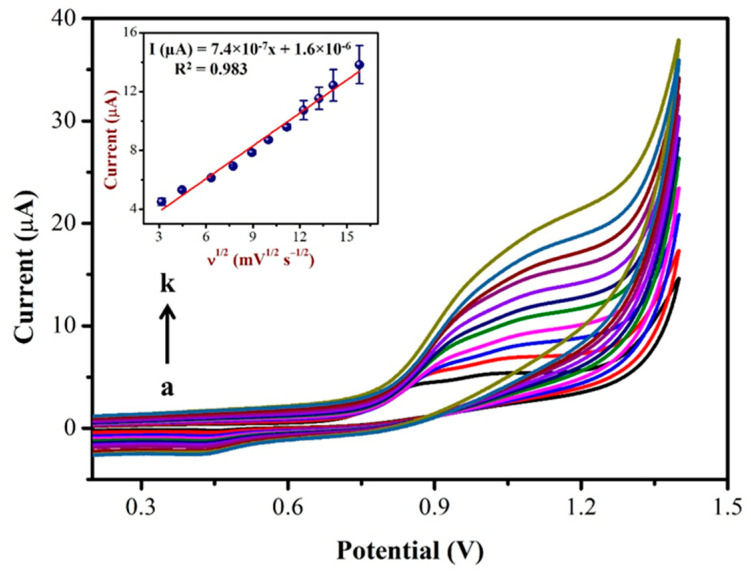
CVs were recorded using AuNPs/Fe(III)Pc/Gr/GCE at different scan rates from (a) 10 mV/s to (k) 250 mV/s in 0.1 M PBS containing 500 µM NIC. Inset: The linear plot was created using oxidation peak current (µA) and the square root of the scan rate (mV.s^−1^). (Error bar indicates the mean value with standard deviation of three measurements, *n* = 3).

Using CV, the impact of the scan rate on NIC oxidation peak currents were investigated in PBS (0.1 M, pH = 7.4) with 0.5 mM NIC as shown in Figure 7. As can be seen, the oxidation peak potential of NIC gradually changed to a high positive potential, indicating a kinetic limit to the reaction [63]. Furthermore, the oxidation peak currents of the NIC increased linearly with the square of the root of scan rate as the scan rate increased from 10 to 250 mV/s (inset of Figure 7).

The linear correlation between the NIC oxidation peak currents with the square of the root of the scan rate (Inset of Figure 7), resulted in a linear equation of Y = 7.4 × 10^−7^x + 1.6 × 10^−6^ and a correlation coefficient (R^2^) of 0.983. Based on the above results, it was confirmed that the oxidation reaction of NIC on the AuNPs/Fe(III)Pc/Gr/GCE was a diffusion-controlled process [64,65].

### 3.5. NIC Detection by Differential Pulse Voltammetry (DPV) 

**Figure 8:**
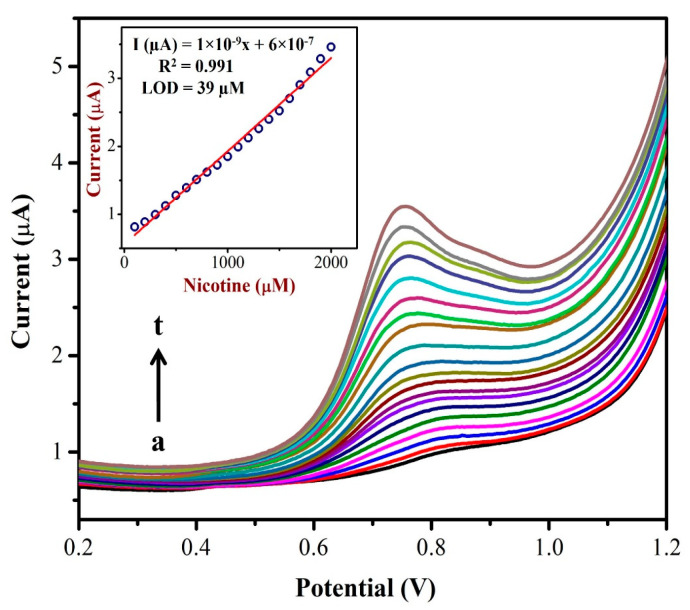
DPVs were recorded using an AuNPs/Fe(III)Pc/Gr/GCE in 0.1 M PBS with the additions of different NIC concentrations from (a) 100 µM to (t) 2 mM. Inset: The calibration plot was made between the NIC concentrations (µM) and oxidation peak currents (µA) of NIC.

The differential pulse voltammograms (DPVs) were recorded using the AuNPs/Fe(III)Pc/Gr/GCE as a working electrode with different concentrations (from 0.1 mM to 2 mM) of NIC as shown in Figure 8. A linear plot was made between the nicotine concentration and oxidation peak currents of NIC as shown in the inset of Figure 8. The NIC oxidation peak currents linearly increased corresponding to the added concentration. A linear regression was identified and the straight-line equation was shown to be Y = 1 × 10^−9^x + 6 × 10^−7^ with an R^2^ of 0.991. The LOD for NIC was found to be 39 µM. The DPV data suggest that the AuNPs/Fe(III)Pc/Gr/GCE could be utilized to detect NIC at a lower potential with a high sensitivity. The high electron density of AuNPs/Fe(III)Pc/Gr/GCE and the phenyl moiety of NIC might have interacted via a π-stacking interaction, which produced the enhanced electro-catalytic effect. The pyrrolidine moiety of NIC undergoes electro-oxidation via. n-methyl hydroxylation (N-MH) [29,66,67].

### 3.6. Effect of pH on NIC Oxidation

**Figure 9:**
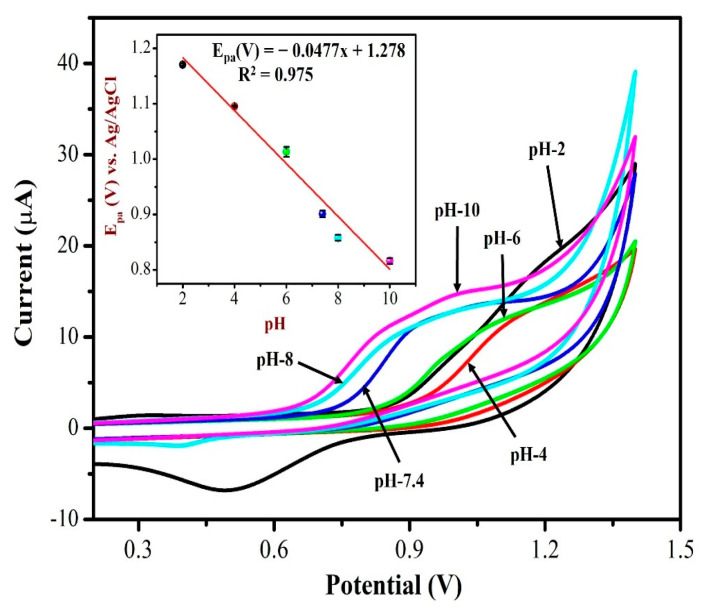
CVs were recorded for 0.5 mM NIC oxidation at a scan rate of 50 mV s^−1^ in 0.1 M PBS with different pH (from pH 2 to 10). Inset: a linear plot was made between the nicotine oxidation potential and pH of the solution. The error bar indicates the mean value with standard deviation of three measurements (*n* = 3).

The electrochemical response of AuNPs/Fe(III)Pc/Gr/GCE towards 0.5 mM NIC was investigated by CV in 0.1 M PBS solution with different pH ranging from 2 to 10. Figure 9 displays CVs of NIC oxidation peak currents and peak potentials in different pH (2–10) solutions. It is worth noting that the oxidation potential of NIC had shifted to negative potential side when the pH was increased from 2 to 10. At lower pH 2, the nicotine oxidation peak was not observed, the CV curve showed only the redox peak of AuNPs. As can be seen, the PBS solution (with pH 7.4 and pH 8.0) showed the oxidation peak of NIC with higher peak currents at AuNPs/Fe(III)Pc/Gr/GCE. At higher pH 8, the peak current of NIC started to reduce a little as depicted in Figure 9. Due to the physiological condition, pH 7.4 was selected as the ideal pH for further experiments. The inset of Figure 9 was also demonstrated the relationship between pH and the oxidation peak potential (E_pa_) of NIC, which resulted with a slope of −47.7 mV/pH, which was comparable to the theoretical value of −59 mV/pH. This proved that an identical number of protons and electrons were involved in the NIC oxidation process on the AuNPs/Fe(III)Pc/Gr/GCE.

A linear graph with an R^2^ value of 0.975 was obtained from the plot of pH vs. NIC oxidation potential as Y = 0.0477x + 1.278 (X = pH; Y = V). The main reason for the high peak current of NIC observed at pH 7.4 may be due to the desirable electrostatic attraction between the NIC and the AuNPs/Fe(III)Pc/Gr/GCE.

### 3.7. Amperometric Determination of Nicotine

**Figure 10:**
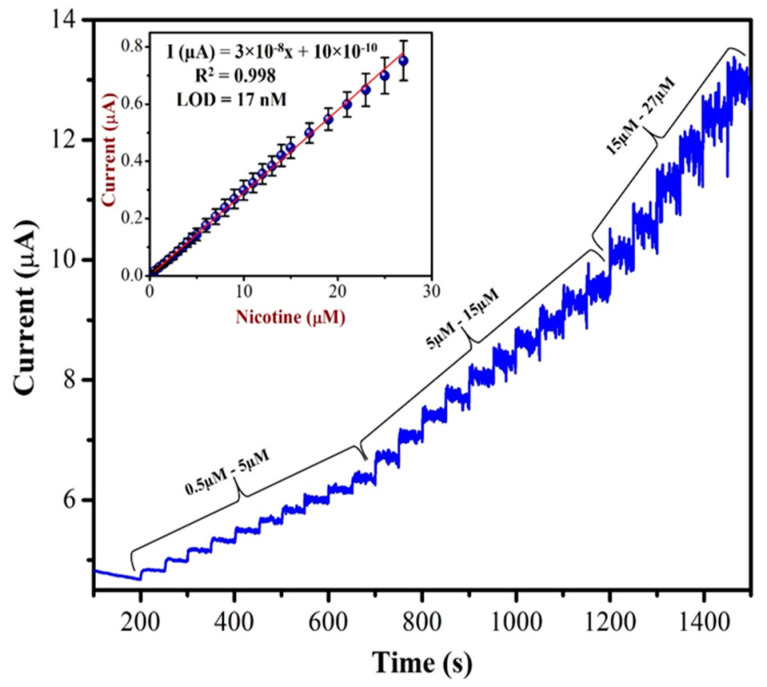
The AuNPs/Fe(III)Pc/Gr/GCE used to record the amperogram (i–t curve) with the additions of various concentration of NIC (from 0.5 µM to 27 µM) in 0.1 M PBS. Inset: calibration curve was plotted between the NIC concentration (µM) and current response (µA). The mean value with standard deviation of three measurements (*n* = 3) was indicated by an error bar.

Amperometric analysis is more suitable for the sensing of targeted NIC compound in a wide linear range. This technique could offer an excellent reproducibility, low limit of detection and high sensitivity [68]. As shown in Figure 10, as-prepared AuNPs/Fe(III)Pc/Gr/GCE was used to record the amperometric (i–t curve) curve for NIC (from 0.5 µM to 27 µM) at an applied potential of +1.0 V in 0.1 M PBS. A linear response was observed for each addition of NIC. However, after the addition of 27 µM of NIC, the sensor response was saturated and started to show a higher noise. The calibration graph was plotted by the data obtained between different concentrations of NIC and the response currents of NIC oxidation. 

The concentration of NIC was increased with subsequent additions. When we double the concentration of NIC, the oxidation currents of NIC also increased linearly. The linear curve was observed with the equation of Y = 3 × 10^−8^x + 10 × 10^−10^ and the R^2^ value of 0.998. The AuNPs/Fe(III)Pc/Gr/GCE’s sensitivity was 0.404 µA µM^−1^ cm^2^ and the LOD was 17 nM for NIC. The analytical effectiveness of the proposed method was also compared with that of other existing NIC sensors. Table 1 shows the comparative analysis between the proposed method and other reported sensors based on oxidation potentials of NIC, LOD, and linear range of detection. It was clear that the AuNPs/Fe(III)Pc/Gr/GCE can be used for the detection of NIC at the lowest LOD compared to other reported sensors.

### 3.8. Interference Study

**Figure 11:**
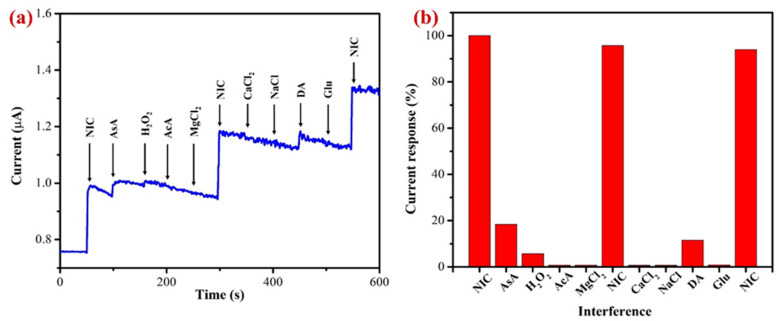
(**a**) The AuNPs/Fe(III)Pc/Gr/GCE used to perform the interference study at 1.0 V in the presence of 10 µM NIC in 0.1 M PBS (pH 7.4) (stirred at 750 rpm) (each interference compound’s concentration was 10 µM). (**b**) A bar graph displaying the percentage of the current response following each addition of interfering chemicals along with NIC (10 µM).

An interference analysis using amperometry (i–t curve) was conducted to confirm the selectivity of the proposed AuNPs/Fe(III)Pc/Gr/GCE as shown in Figure 11a. With the addition of possible interfering substances, the amperogram was recorded in PBS solution (0.1 M, pH 7.4) containing 10 µM of nicotine at an applied potential of +1.0 V and the electrolyte was stirred at 750 rpm. In accordance with the findings, acetic acid, magnesium chloride, calcium chloride, sodium chloride, and glucose did not interfere or produce any signals (Figure 11b).

It is worth noting that the ascorbic acid, hydrogen peroxide, and dopamine were slightly interfered and produced a minor response. All the interference compounds were taken by 1-fold concentration. These compounds were chosen for the selectivity investigation due to their broad availability in real-world samples such as human bodily fluids [73,76]. Additionally, the effects of other alkaloids found in tobacco were not examined because of their negligible concentrations (0.2–0.5%), which might not have an impact on the determination of NIC [19,70,77]. Our results demonstrated that these tested compounds did not interfere significantly, which was most likely caused by the fact that interferents’ oxidation or reduction potentials were not matched with the oxidation potential of NIC. This study indicated that the proposed sensor was selective towards NIC in physiological conditions.

### 3.9. Detecting NIC in Saliva Samples

**Figure 12:**
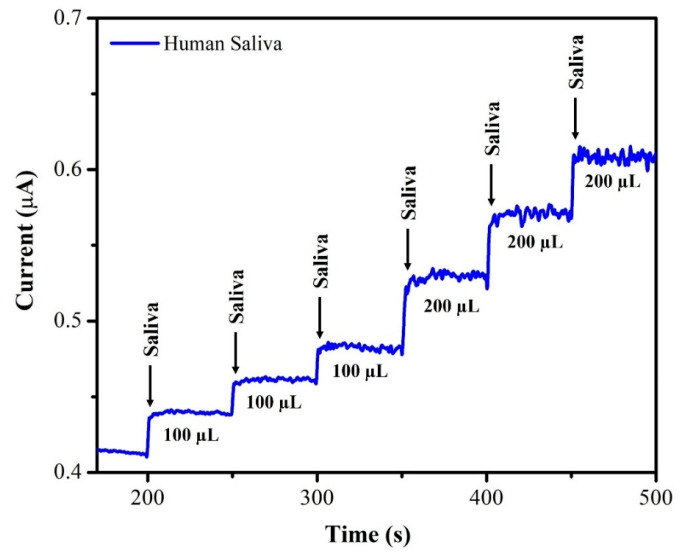
Amperogram was recorded using AuNPs/Fe(III)Pc/Gr/GCE with NIC in saliva sample at +1 V in 0.1 M PBS (pH 7.4). The solution was stirred at 750 rpm.

**Figure 13:**
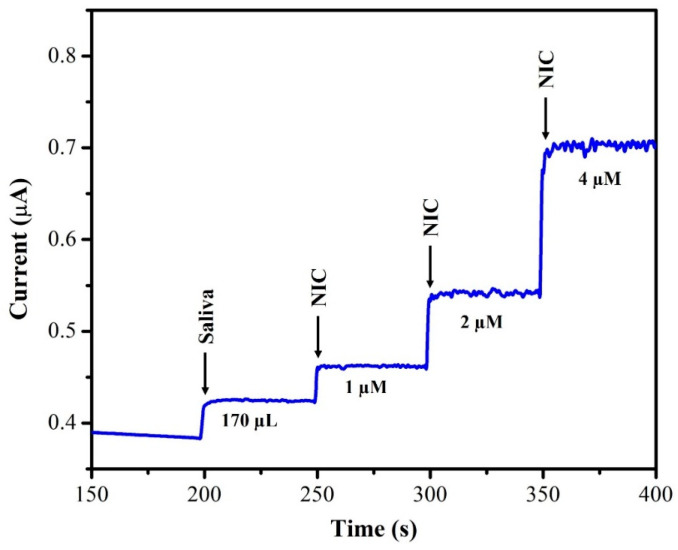
i–t curve was recorded using AuNPs/Fe(III)Pc/Gr/GCE in saliva samples with various concentrations of standard NIC solutions added into 0.1 M PBS (the rotation rate was 750 rpm).

The unknown concentration of NIC was determined using the amperometry technique with each sample undergoing three separate measurements (*n* = 3), and the average of the three readings being displayed. The supernatant of the saliva sample was diluted with DI water at a 1:1 ratio. From this saliva solution, 100 µL of the sample was injected into 20 mL of 0.1 M phosphate-buffered solution (pH = 7.4) and the oxidation current response was recorded using AuNPs/Fe(III)Pc/Gr/GCE. After each addition of a saliva sample, the current response of the sensor increased, as depicted in Figure 12. The recovery investigations were also performed on a sample of human saliva as shown in Figure 13. First, 170 µL of saliva was combined with 0.1 M phosphate-buffered solution (pH = 7.4), then standard NIC was added, and finally, three different NIC concentrations (1 µM, 2 µM, and 4 µM) were spiked into the mixture. To quantify the unknown concentration of NIC in the saliva sample, the amperogram was recorded using the AuNPs/Fe(III)Pc/Gr/GCE at +1.0 V. The linear calibration curve was then used to determine the unknown concentrations. Table 2 shows the estimated NIC concentration in the sample of human saliva as well as in the spiked standard NIC solutions, demonstrated the successful recovery of spiked NIC in the salivary samples (95.8–101.8%).

#### Repeatability and Reproducibility Studies

**Figure 14:**
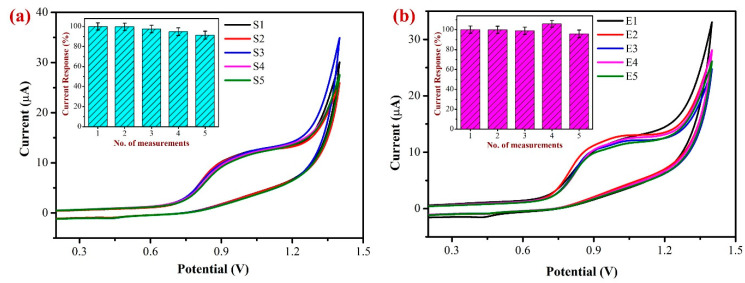
(**a**) Repeatable determinations of 500 µM NIC were performed in 0.1 M PBS (pH 7.4) using the same AuNPs/Fe(III)Pc/Gr/GCE for five repeated measurements by CV. (Inset: the bar graph showed the no. of measurements vs. the NIC oxidation peak current responses after each experiment). (**b**) Reproducibility test was performed with 500 µM NIC using five independent AuNPs/Fe(III)Pc/Gr modified GCE’s in 0.1 M PBS at a scan rate of 50 mV/s. (Inset: the bar graph shows the no. of measurements vs. the NIC oxidation peak current response after each measurement and the standard deviation was provided).

In order to determine the repeatability of the sensor and examine the stability of the modified electrode, the oxidation of 500 µM NIC in 0.1 M PBS at a scan rate of 50 mV/s was carried out using the freshly developed AuNPs/Fe(III)Pc/Gr/GCE (Figure 14a). The inset of Figure 14a exhibits the bar diagram representation of repeatability data of five consecutive measurements. During the first three independent measurements, the I_pa_ of NIC showed slight variations from 1 to 5% at +0.85 V. After that, around the fourth and fifth successive readings, the I_pa_ of NIC began to decline, probably as a result of some coated material starting to leach out of the GCE after the third measurement. Despite this, the decline in I_pa_ was only about 5–7% when compared to the first measurement, demonstrating that the AuNPs/Fe(III)Pc/Gr/GCE seems to be stable for repeated measurements. These findings further supported that the AuNPs/Fe(III)Pc/Gr/GCE can be used for repeated NIC measurements.

To investigate the sensor’s repeatability and accuracy, 500 µM NIC in 0.1 M PBS was oxidized at a scan rate of 50 mV/s using five newly prepared individual AuNPs/Fe(III)Pc/Gr modified electrodes (as shown in Figure 14b). The bar diagram demonstrates the reproducibility measurements of NIC oxidation utilizing the five separately prepared AuNPs/Fe(III)Pc/Gr/GCE’s. The purpose of this study was to assess the consistency and reliability of the modified electrode in the determination of NIC. Following the examination of the five individually modified electrodes, the I_pa_ of NIC exhibited a minor variation at +0.85 V. However, all the five modified electrodes’ average current response was determined to be 95.6%. These findings indicated that the proposed electrochemical sensor (AuNPs/Fe(III)Pc/Gr/GCE) could be prepared and used for NIC detection with a high reliability.

## 4. Conclusions

A modified glassy carbon electrode with a AuNPs/Fe(III)Pc/Gr nanocomposite had been successfully developed for NIC analysis. The FESEM, EDS spectrum and E-mapping results confirmed the incorporation of Fe(III)Pc and AuNPs into the nanocomposite with Gr. This new sensor showed a substantial oxidation peak current for NIC at a lower potential. The AuNPs/Fe(III)Pc/Gr nanocomposite-based sensor showed a lowest LOD of 17 nM with a higher sensitivity toward NIC. The AuNPs/Fe(III)Pc/Gr nanocomposite-based sensor was also used for the selective detection of NIC in the presence of interfering organic and inorganic compounds in PBS (0.1 M, pH 7.4). Moreover, the repeatability and reproducibility results showed that the AuNPs/Fe(III)Pc/Gr nanocomposite-modified electrode had a greater stability and a high accuracy. Additionally, the recovery of NIC was computed and the NIC content in a human saliva sample of a smoker was evaluated using a AuNPs/Fe(III)Pc/Gr based sensor in 0.1 M PBS. Finally, we propose that AuNPs/Fe(III)Pc/Gr nanocomposite would be a viable material for the fabrication of sensors to determine NIC levels in important real-world samples.

## Figures and Tables

**Table 1:** The analytical comparison between various NIC electrochemical sensors and proposed method.

Electrode	Catalyst	Electrolyte	Epa (V)	Technique	Linear Range (µM)	LOD (µM)	Test Sample	Reference
GCE	TiO_2_/PEDOT	PBS, pH = 7.4	0.88	AMP	0–5000	4.9	–	[1]
GCE	AgNPs	PBS, pH = 7.4	0.9	AMP	2.5–105	0.135	Human saliva	[23]
GCE	MXene/PHC	PBS, pH = 7.4	1	CV/AMP	0.25–37.5	0.027	Human sweat	[24]
BPPGE	MWCNT	PBS, pH = 8	0.65	CV	0–1000	1.5	–	[25]
GCE	MWCNT/ACS	PBS, pH = 8.0	0.65	AMP	5–1395	1.42	–	[69]
GCE	MWCNT	Na_2_C_2_O_4_, pH = 4.5	1.4	DPV	31–1900	9.3	Cigarettes	[70]
GCE	P-AHNSA	PBS, pH = 7.5	0.88	SWV	1–200	0.866	Cigarettes	[71]
PGE	SDS (surfactant)	PBS, pH = 7	0.84	SWV	7.6–107.5	2	Cigarettes	[72]
A-GCE	–	PBS, pH = 7	0.83	SWV	1–200	0.7	Cigarettes	[73]
CPE	TiO_2_	BRB, pH = 5	0.87	CV/DPV	2–540	0.0134	Cigarettes/Urine	[74]
SPE	CNC	PBS, pH = 7	0.75	CV	10–1000	2	Artificial saliva	[75]
GCE	AuNPs/Fe(III)Pc/Gr	PBS, pH = 7.4	1.0	AMP	0.5–27	0.017	Human saliva	Current work

Footnotes: E_pa_—oxidation or anodic peak potential, I_pa_—oxidation or anodic peak current, LOD—limit of detection, GCE—glassy carbon electrode, BPPGE—basal plane pyrolytic graphite electrode, PGE—pencil graphite electrode, A-GCE—activated glassy carbon electrode, CPE—carbon paste electrode, SPE—screen printed electrode, TiO_2_/PEDOT—titanium dioxide/poly(3,4-ethylenedioxythiophene), MXene/PHC—MXene/palladium hydroxide-supported carbon, MWCNT/ACS—multiwalled carbon nanotube/alumina-coated silica, P-AHNSA—Poly (4-Amino-3-Hydroxynaphthalene Sulfonic Acid), SDC—anionic surfactants, CNC—carbon nanotube cluster, BRB—Britton–Robinson buffer solution, PBS—phosphate-buffered solution, Na_2_C_2_O_4_—sodium oxalate buffer, CV—cyclic voltammetry, DPV—differential pulse voltammetry, AMP—amperometry, and SWV—square wave voltammetry.

**Table 2:** Determination of NIC concentration in human saliva sample using a AuNPs/Fe(III)Pc/Gr/GCE sensor.

S. No.	Samples	Added (µM)	Found (µM)	Recovery (%)	SD	RSD %
1	Human saliva	-	1.00	-	0.014	1.403
2	Std NIC	1.00	1.95	95.8	0.004	0.439
3	Std NIC	2.00	2.97	98.5	0.016	0.846
4	Std NIC	4.00	5.07	101.8	0.033	0.827

## Data Availability

Data is available upon a reasonable request.
